# Developing a model of help giving towards people with a mental health problem: a qualitative study of Mental Health First Aid participants

**DOI:** 10.1186/s13033-018-0228-9

**Published:** 2018-08-21

**Authors:** Alyssia Rossetto, Anthony F. Jorm, Nicola J. Reavley

**Affiliations:** Population Mental Health Group, Melbourne School of Population and Global Health, The University of Melbourne, 207 Bouverie Street, Carlton, VIC 3010 Australia

**Keywords:** Mental health first aid, Helping behaviours, Thematic analysis, Mental health problem, Informal supports

## Abstract

**Background:**

Members of the public frequently perform mental health first aid actions in daily life, and people with mental health problems often cite informal supports as motivators of professional treatment seeking. However, a thorough understanding of how, when and why these actions are undertaken is lacking. This research aimed to investigate the helping experiences of community members trained in Mental Health First Aid, understand the factors that might facilitate and deter helping behaviours, and develop a preliminary model that outlines the process of providing help to someone with a mental health problem.

**Methods:**

Community members who had received Mental Health First Aid training (*n* = 16) were recruited from an urban Australian university and completed an in-depth, semi-structured interview about their experiences of helping someone with a mental health problem. Data were analysed using thematic analysis. Member checking was used to assess the accuracy and transferability of the findings.

**Results:**

The results suggested that several common elements were present in participants’ narratives, including recognising a recipient in distress, considering reasons to intervene or not, choosing a course of action, and noting the outcomes of help. These themes were collated to form the main stages of a preliminary model of helping.

**Conclusions:**

The findings of this study highlight the many considerations involved in deciding whether and how to assist a person with a mental health problem, and the complex, dynamic nature of the helping process itself. The preliminary model of helping may be used to enhance the content of educational programs and public health messages.

## Background

A mental health problem affects a person’s thinking, emotions and behaviour, disrupting their capacity to work, perform daily activities and engage in satisfying personal relationships [1]. When these symptoms reach clinically significant levels, the person can be diagnosed with a mental illness. Approximately 29% of people worldwide aged 16–65 have met criteria for a mental illness in their lifetime [2], and this significantly increases their risk of dying by suicide [3]. However, research suggests that a significant proportion of adults meeting the criteria for a mental illness do not seek treatment or use available mental health services [4]. Delaying treatment seeking adversely affects future recovery prospects [5, 6] and quality of life.

Members of the public consistently endorse informal supports (e.g., family and friends) as helpful when addressing mental health concerns [7, 8]. Furthermore, people with mental health problems often nominate the influence of members of their social networks as a key reason for seeking professional help [9, 10]. This suggests that informal supporters are well placed to practise mental health first aid [1], which includes recognising symptoms or behaviours indicative of a developing mental health problem, offering assistance and providing support during treatment and recovery.

A small group of studies has investigated what types of help members of the public offer, or intend to offer, to someone experiencing a mental health problem. The type and quality of help provided is often associated with particular participant characteristics, such as female gender [11], knowledge, beliefs or attitudes about mental health problems [e.g., stigma; 12, 13] and other variables, such as prior psychiatric history [14]. This research improves understanding of what actions are most often practised, and who may benefit from more information and education about mental health problems. However, it provides little insight into how, when and why people decide to provide help to individuals experiencing mental health problems.

Understanding why informal supporters choose to assist people with mental health problems would be useful for several reasons. Determining whether a decision-making process for helping someone with a mental health problem exists, and what stages or components are involved, would provide insight into the thoughts and feelings that inform the behaviour of informal helpers. This information could be used by community education programs which equip people with the knowledge and skills to offer appropriate and timely assistance. It could also establish how well knowledge translates into action, thus acting as a gauge for the effectiveness of existing education programs and public health campaigns. Identifying what factors motivate and deter offers of assistance would help to establish whether and how deterrents can be modified in ways that encourage people with mental health problems to seek help and ensure they are adequately supported during this process. For example, lacking knowledge of how to help is likely to inhibit helping, and research suggests that education programs such as Mental Health First Aid [MHFA; 15] improve both knowledge about and rates of helping behaviour [16]. MHFA is a program which educates community members on how to provide help to a person developing a mental health problem or mental health crisis until the crisis resolves or appropriate professional help is received, using an action plan comprised of five steps: approach, assess and assist with crisis, listen non-judgmentally, give support and information, encourage appropriate professional help and encourage other supports [1].

Lastly, this research may help to inform the framing of messages aimed at increasing rates of helping in the community. Previous research [17] suggests that public education campaigns which emphasise simplistic, rational models of behaviour (e.g., “If you see this, you should do that”; p. 6) do not accurately reflect the reality of approaching and assisting someone with a mental health problem. Rather, interviews with people bereaved by suicide suggest that the decision-making process surrounding the provision of help is complicated, highly emotional, and influenced by a variety of respondent and recipient factors [17], including a lack of explicit warning signs, a failure to acknowledge the seriousness of the situation and inadequate communication with the suicidal person about their mental health. This study is notable for being one of the first qualitative investigations for exploring potential helpers’ actual behaviours and for providing important insights into the factors that can influence helping behaviour in a mental health crisis. However, it is uncertain whether these factors operate across other mental health conditions, what factors facilitate (rather than inhibit) helping behaviours, or what the effects of help are for helpers and recipients.

The present study seeks to explore these aspects in greater detail by interviewing a sample of adults who had participated in a MHFA course. MHFA course participants were chosen for this research because, as a result of their training, they had the knowledge to correctly recognise symptoms and behaviours indicative of an established or developing mental health problem, which facilitated the discussion of examples relevant to the objectives of this research. These participants could also offer a more informed reflection of their helping experiences both before and after their MHFA training that may not necessarily have been acquired from totally naïve participants, and they could articulate whether and how the training affected their helping attitudes and behaviours. This research aims to gather comprehensive information about MHFA trained community members’ experiences of helping, understand the factors that facilitate and deter helping behaviours and develop a preliminary model that outlines the process of providing help to someone with a mental health problem.

## Methods

### Procedure

Ethics approval was received from the University of Melbourne’s Human Research Ethics Committee. Participants were recruited and interviewed between February and April 2014. Respondents who had seen advertisements placed at the University of Melbourne contacted the researchers by email to express their interest in participating. They were interviewed if they met the following criteria: aged over 18, had completed any MHFA course at least 3 months prior to the interview, were not mental health professionals, and consented to the interview being recorded and transcribed. Participants were interviewed at the university, as they were generally familiar with the location and the interviews could be recorded without excessive background noise or interference [18].

Participants first read and signed the information and consent form and completed a brief questionnaire asking for demographic information and details about the MHFA course they completed. They then participated in a semi-structured interview, which asked questions about their helping experiences towards a person with a mental health problem (including times when they had and had not assisted), what considerations influenced their helping behaviours and what the outcomes of their decisions were for the helper and the recipient of aid. Interviews lasted between 36 and 88 min (mean = 68.62 min).

Upon completion of the interview, participants were presented with a $20 gift voucher and told that they would be emailed a transcription of the interview to look over and comment on. Recruitment continued until a sufficiently diverse range of helping and non-helping situations had been described by participants and saturation (defined as the point where interviews no longer elicited unique themes) was achieved [19].

Interviews were audio recorded and transcribed using an orthographic (verbatim) style into a word document within 3 weeks of completing the interview and checked for accuracy by the interviewer. Any questions that the interviewer subsequently had for the participant after reflecting on the content of the interview were included in comment boxes alongside the transcription. Participants were emailed a copy of their interview and asked to answer any questions, provide additional comments as they saw fit and reaffirm their consent for the data to be used in the research. Fourteen participants provided feedback. The transcribed interviews, plus the comments provided post-interview, were included as data in the analysis. Identifying data were removed from the transcripts prior to analysis.

In addition to member checking by the study’s participants, rigour was also sought by presenting this research at an annual conference of MHFA instructors in September 2015. The presentation was followed by a feedback session with instructors to obtain their thoughts on the model and its relevance to them and the MHFA course.

### Data analysis

Thematic analysis was used to analyse the data [20]. Although this study was not driven by any particular theoretical position, given that there is no overarching theory of helping behaviour towards people with a mental health problem, the interview questions were developed to explore particular experiences and motivations around helping someone with a mental health problem. Data analysis was therefore conducted in an inductive fashion, according to the process and quality guidelines provided by Braun and Clarke [20]. Initial data coding was undertaken during transcription, with a second and third coding run occurring after all the interviews were transcribed and comments had been received from participants. One researcher coded all of the data. Data items (codes, themes and sub-themes) were organised into lists, tables and thematic maps. Attention was focused on understanding perceived patterns and inconsistencies in the data, discovering where participants’ narratives converged and diverged, and noting if and how the interviewer influenced and interpreted the participants’ responses during the interview and the coding process.

## Results

A purposive sample of 16 adults who had completed MHFA training was recruited. The characteristics of the sample relevant to the research are presented in Table 1. Participants are referred to by their participant number throughout this paper. Participants were aged 20–62 years (mean = 30.75 years, standard deviation = 10.43, median = 28.50 years), with 12 females and 4 males interviewed. These numbers approximate the ratio of females to males participating in MHFA courses, where females routinely constitute 65–85% of participants [21–25]. Most participants had a personal or professional interest in health or mental health, and two had experienced mental health problems themselves. Those with a professional interest in health or mental health worked as academics, worked for an organisation with a focus on mental health (e.g., a charity), or held a position which involved a consideration of health concerns in others (e.g., an occupational health and safety officer). Participants had held their MHFA certificates for between 1 and 8 years (mean = 2.87 years).

**Table 1:** Participant reference numbers and characteristics

Participant number	Gender	Year of MHFA course completion
P1	M	2012
P2	F	2007
P3	F	2006
P4	F	2013
P5	F	2012
P6	F	2013
P7	M	2011
P8	F	2012
P9	F	2011
P10	F	2011
P11	F	2012
P12	F	2011
P13	M	2012
P14	M	2013
P15	F	2011
P16	F	2011

**Fig. 1:**
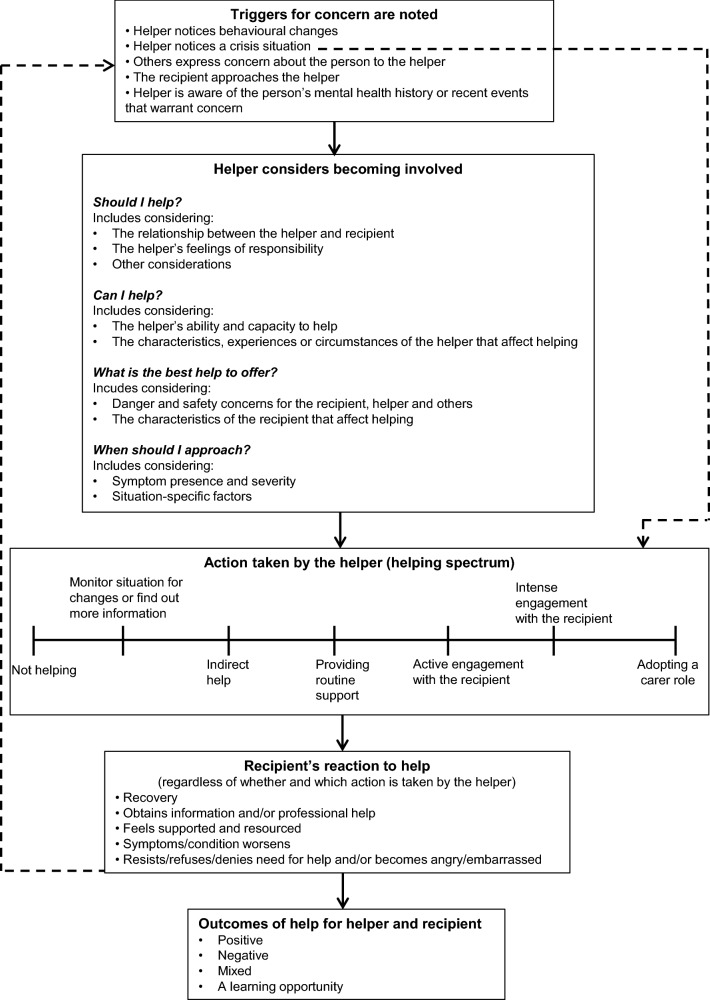
Model of help giving for situations involving a mental health problem or mental health crisis

Participants mentioned a total of 55 examples of situations they had been in where a person was experiencing a mental health problem: 16 (29.1%) had occurred before undertaking the MHFA course, 25 (45.5%) had occurred after completing the course, nine were ongoing (they had begun before the course and had continued through to the present; 16.4%) and the timeframe of five examples was not specified (9.1%). Thematic analysis suggested there were several common elements and stages underlying participants’ experiences. Noticing or being told of a recipient in distress, considering reasons to intervene or not, choosing a course of action, and, where possible, noting the short- and long-term outcomes of help for the helper and recipient, were present in almost every narrative. These themes formed the main stages of a preliminary model of helping, depicted in Fig. 1, and are detailed in the following sections.

### Triggers for concern

The helper often noticed behavioural changes indicating a developing mental health problem, or the beginning of a mental health crisis (e.g., suicidal thoughts, non-suicidal self-injury, aggressive behaviour). Occasionally, the helper was aware that the recipient might be at risk and was already monitoring for emerging signs of a mental health problem or mental health crisis. Helpers were also occasionally told by someone else about the recipient’s problems, for example, P3 mentioned that *“…another friend had sort of flagged it, but I also knew that perhaps they probably couldn’t do much themselves, either.”* Lastly, recipients themselves sometimes approached the helper to discuss their problems.

### The helper considers becoming involved

All participants gave reasons for helping or not helping in particular situations, and discussed what would motivate or deter them more generally. When considering their potential involvement, participants often spoke in terms of whether they “could” or “should” help. They also discussed practical details like appropriately timing their offers of help and what help would be most useful for the recipient. Participants seemed to query four aspects of their potential involvement before moving into the help provision stage:

- Should I help?
- Can I help?
- What is the best help to offer?
- When should I approach?

These questions form the major themes of this section, under which sub-themes related to reasons for or against helping are discussed in more detail. Although some participants tried to establish which reasons were the most influential in making their helping decisions, very few were clear on how precisely their reasons interacted, or which took prominence at what times.

#### Should I help?

This involved three main considerations: the nature of the relationship between the helper and the recipient, the helper’s feelings of responsibility, and other factors that may affect the decision to offer help.

#### The relationship between the helper and the recipient

This was an important consideration for the majority of participants and referred to the type and characteristics of the relationship between the helper and recipient that affected helping. Helping situations frequently involved recipients who were friends, family members or intimate partners, and participants cited the close nature of these relationships as a facilitator. However, how participants thought that this influenced their helping varied, with some stating that the inherent affinity of the relationship itself was the motivator (e.g., caring about the recipient) and others describing a specific characteristic of the relationship as taking precedence at the time (e.g., having provided help in the past to a recipient, or reciprocating previously provided help). Some participants said that sometimes they felt like they had no choice but to help someone close to them. Others said that they were the best person to help at the time, or that other potential helpers were unavailable to assist.

Conversely, limitations in the relationship could deter helping. One common reason for refusing to help was because the helper did not know or was not close to the potential recipient. Helpers sometimes felt that it was “not my place to help”, although what this meant was not always clear. Occasionally it was suggested that other people had a closer relationship with the recipient, were better qualified to help, or could provide more capable help that would be better received by the person.

Helping a particular recipient might also be difficult, awkward or uncomfortable for both parties. P11 said of one recipient, *“It’s an active rejection. Um, I can talk about physical things, ‘How’s your knee?’…but I can’t talk about the other things…the barriers are up and so we, we don’t go there.”* Several participants noted that their help might not be wanted or welcome. Participants also remarked that they might not be the recipient’s preferred source of help.

Believing that role confusion might occur was also discussed, and was related to participants’ past experiences with helping a recipient to the extent that they effectively became carers. Helpers did not want this repeated, explaining their subsequent reluctance to help if the situation was ambiguous, if the expected roles and depth of involvement were not clearly defined at the outset, and if they were unsure whether the recipient would become over-reliant on them. P5 explained this by saying of a former housemate, *“…she wasn’t doing much for herself, so…even if she’s going through difficulty now, I wouldn’t put myself back in that situation to help her…she was seeing a counsellor for quite a while, and she was able to explain her feelings for a long time just as friends, but it was when it was no longer in sort of a friendship way, it was needing us, like the household and friends to…constantly assure her that people aren’t against her or not really being able to invite people to the house that she didn’t like, that it really affected our lives and it wasn’t about just being a friend and support to her anymore… it then became too big a task, it was then being able to assert that I can’t help, or we can’t help.”*

Two other relationship-based reasons were mentioned. P9 discussed having expected, but not received, help from her recipient in the past as a reason for considering ending a helping relationship (ultimately she did not do this). P16 said that she had decided not to offer help to someone because she and the potential recipient had extremely contrasting attitudes and personalities, which she felt would limit her ability to help constructively.

#### The helper’s feelings of responsibility

Fourteen participants mentioned feeling a sense of responsibility to help someone. Responsibility was related to the relationship-based reasons for and against helping, as some participants framed their helping in terms of feeling an obligation to the recipient because of the relationship they shared. P4 and P12 explained feeling responsible *for* their recipients as a reason for assuming larger than anticipated helping roles. This seemed linked to both the severity of their recipients’ mental health problems and the particular dynamics and expectations present in these relationships. P4 said, *“I had someone who was dependent upon me, like, totally dependent, and as much as I want to pretend like that was hard, it was also…comforting? In a way…it was like having someone, like a pet, you’re having someone be like, ‘What do I do? Be there for me, love me.’…that responsibility really weighed me down a lot, and it made me feel a lot older and a lot more pressure than I needed…that was really difficult, to feel like a lot of the time, a lot of things were dictated by what emotion she was in that day.”*

Participants also discussed times where they had a duty of care to a recipient as part of a professional or volunteer role (e.g., P11 as a teacher, P2 as an obstetrician, P6 as a telephone counsellor); this sometimes led them to help when they may not have done so otherwise. Some participants, who did not have a formal role or duty of care, felt a sense of responsibility because they believed that no-one else would help the recipient.

It was also possible for helpers to want to avoid assuming responsibility for a recipient, or to feel that it was the responsibility, role or duty of other people to help instead. P10 framed her non-involvement in workplace situations in terms of not wanting to assume responsibility for helping and concurrently believing that others should adopt a helping role. She said*“…it is a barrier for people in a workplace, because you wouldn’t mind it so much if it’s a friend or a family member, you’re like, “I want to share this with you, I want to help,” but at work, you’ve got your own work to do, and if anything is going to be an addition to that load, unless it’s not, if it’s not directly affecting you, if it’s not your boss, or it’s not your, someone you supervise, then really, is it going to be an added burden to you, and are you actually going to help them much?”* P10 also believed that it was difficult to discuss mental health problems pragmatically and without offending the recipient in the workplace compared to elsewhere. Other participants spoke about not wanting to involve themselves in situations where they risked becoming the main or only helper.

#### Other considerations

This sub-theme comprised reasons that were unique to particular helpers, recipients or situations. Some participants thought that offering help would unintentionally result in poor outcomes for the recipient or other people. P9 explained that if helping her severely depressed friend began affecting her partner’s or housemate’s lives, she would have to rethink her helping: *“And it’s not about them being more important than her, it’s more about, they also have the right for this to not be impacting their life in a really negative way.”* There was also a perception that help might not be helpful for the recipient, for example, if it exacerbated the situation, did not make a difference or drew unwanted attention to the problem or the person.

Four other reasons were mentioned that affected helpers’ perceptions of whether they should become involved in an unfolding mental health first aid situation. P9 recounted a time when she stepped into relieve another helper, providing the recipient with an alternative source of help. P14 stated that he would occasionally help if he believed he could bring something to the situation that had not previously been offered. P12 believed that any acknowledgement of or assistance in a mental health first aid situation was better than no intervention. P1 and P4 believed that by helping particular people, they were also helping themselves. For example, when helping her partner with her suicidality, P4 was motivated by a fear of losing her partner and their relationship; by helping her she could retain what they shared.

#### Can I help?

Concerns in this theme included the helper’s ability and capacity to help, and the characteristics, experiences or circumstances that affected their helping.

#### The helper’s ability and capacity to help

Almost every participant mentioned the importance of having the knowledge, skills, time, physical and emotional resources and confidence to help a recipient. P9 summarised this by explaining, *“I feel like I have the skill set to do it, and I have the means to do it. It’s not like I’m juggling…care requirements from my family’s perspective…I know when she’s pushing some of my boundaries, I know when I need a break, I know some of the things that work for me, I know that if I really wanted to I could access* [professional] *supports and have someone provide me advice and things like that. So, yes, I do feel* – *I may not always be thrilled about it, but I do feel like I have the skills to do it.”* Feeling capable of helping suggested that participants believed they could address the situation effectively, that their actions would be helpful and that they could make a difference to the recipient or their situation. Having the time and resources to assist someone was also important; participants were likely to help if they were available, or if it did not take long to help. Similarly, some participants mentioned feeling capable of helping because they were socially supported. For example, P10 said that during her encounter with a stranger experiencing delusions and paranoia at a public pool, she felt an added sense of safety and confidence because her partner was also present and helping the person.

Participants also explained that they were less likely to help when they perceived their abilities or capacities to be diminished in some way. The most often cited barriers were lacking the time or skills, needing to self-care, and lacking confidence. Other reasons included feeling overwhelmed or out of one’s depth (especially if the helping situation occurred unexpectedly), having more important issues to address or cope with at the time, already having carer responsibilities and perceiving that *“you can’t always help everyone”* (P3). If participants did not feel capable of helping at the time, they would try to source other help for the person, for example, by giving them the details of a counselling service (P13) or asking others to assist the recipient or the helper (P12). Helpers may also be genuinely unable to properly care for a recipient. P15 described how her family were forced to realise that her brother’s schizoaffective disorder was better managed onsite in a rehabilitative program than at home. She said, *“…my nonna* [grandmother] *has this expectation that she’ll* [P15’s mother] *look after my brother through thick and thin, and actually she’s changing her tune, though, now…you know, maybe it’s not the best context for him to be in that home environment* – *it’s not making him better, so maybe he does need to experience something different, meet some other people and negotiate life.”*

#### The characteristics, experiences or circumstances of the helper that affect helping

This encompassed how the helper perceived themselves, or how they interpreted their experiences. Personality characteristics such as sociability, empathy or a proactive nature, were perceived to facilitate helping, whereas traits such as shyness were believed to negatively affect the likelihood or effectiveness of helping. Age and experience was another attribute that affected helping, with P8 and P14 both mentioning two long-term helping situations that they could not assist with when they first encountered the recipient because they were too young to offer help; they assumed helping roles as they aged. Most participants believed that there was a moral imperative to their offers of assistance, or that helping embodied a personal value. Wanting to help others was often mentioned, implying altruistic or prosocial motivations. P3 alluded to reciprocity and karma, saying, *“…if I was in that position, I would appreciate someone doing something.”*

Family and cultural values were significant influences for P8, P9 and P15. P9 came from a family with large social networks and helping was an important and normal aspect of her childhood. P15 discussed feeling pressured by her Italian family’s expectation that she assume a caring role for her brother with schizoaffective disorder. P8 and P11 described offering help if other people were also helping. P8 said, *“I’m not always the first to act…Looking at the group dynamics, I think that’s what I do more often.”*

Particular experiences were also mentioned as having shaped participants’ perspectives on helping. P16 explained that she had once been involved in an abusive relationship with a person with severe mental health problems, where she learnt *that “…a lot of it is about drawing the lines between what you can and can’t help with and what’s your stuff and what’s somebody else’s stuff. And typically in a situation like that, those lines get very, very, deliberately blurred, and it takes a while to unwind all that.”* After leaving the relationship she realised that *“…a lot of what used to motivate me to help was actually the gratitude that would be given in return… I would help* [people] *so that they would look up to me. But, as I’ve gotten to a place in my life where I have far greater respect for myself and for my own life, I actually don’t need that from other people anymore. So my helping behaviour has changed, and it’s also actually become a lot more confined. So rather than wanting to help pretty much anybody who comes into my path that has any kind of problem…I’m much more, now, only inclined to extend myself for those who are close in my circle.”*

Perceiving a lack of social or emotional support from significant others was discussed by P3 and P9; helping was easier for them when they could talk about the situation to someone who was level-headed or less involved. Participants also explained that occasionally they did not want to help, because they did not want to intrude or because they were not asked to help.

#### What is the best help to offer?

This theme encompassed two concepts: danger and safety concerns for the recipient, helper and others, and the characteristics of the recipient that affect helping.

#### Danger and safety concerns for the recipient, helper and others

Almost all participants cited this sub-theme in some way. Danger and safety concerns included three ideas: that harm might occur to the recipient or others if the helper did not intervene; whether the helper thought it was safe for them to assist; and that the recipient’s symptoms were severe or concerning enough to warrant intervention. Commonly stated reasons for helping included noticing a developing or escalating crisis situation, resulting in a high probability of danger or harm to the recipient or others, or helpers believing there was a low level of danger to themselves in a given situation. Conversely, being unsure of their own safety acted as a deterrent. Some respondents linked this to the severity of the recipient’s problems, or the unpredictability of their behaviour. For example, P14 stated, *“…if there was an obvious threat of danger, I might not be so likely to get involved, and that’s not a, ‘People with mental health problems are dangerous,’ but it’s more of a kind of* - *if there’s a drunk dude with a broken bottle, I’m probably not going to help out, so maybe perceived dangerousness of the situation might come into it a little bit, or self*-*protection kind of thing.”* Few participants could specify how severe a mental health problem or a situation had to be before they decided not to help, or at what point they felt there was sufficient danger to themselves. P16 explained the complexities of being in an intimate relationship with a person with multiple severe mental health problems. She said that the realisation that this was *“not a bearable or a safe place for me to be in”* was very gradual, and extricating herself from the situation, although she was acutely aware of the danger to herself, was difficult. Decisions around perceived danger and safety seemed context dependent and complex to make.

A related concern was feeling scared or intimidated (common examples included people who may be acting under the influence of drugs or alcohol or were bigger or stronger than the participant). One participant explicitly stated feeling scared of people with mental health problems as a result of previously held stigmatising attitudes, which used to preclude her intervening.

While most participants said that a crisis situation would increase their motivation to help, a minority stated that they were not comfortable or confident intervening in these situations. During a crisis they would attempt to help indirectly by, for example, calling the police or referring the person on to better qualified help, and try to minimise their own involvement. P12 said, *“I’m not sure I would be able to help in terms of if they’re going through a breakdown, or if they’re feeling really suicidal. Because, I’m not sure if, I can be very emotionally…objective, or if I can do it without my emotions getting in the way and that sort of thing…I’d probably ask for help…if it’s out of my control, then I think* – *out of my capabilities* – *then I probably would just leave it to professionals.”*

#### Characteristics of the recipient that affect helping

This sub-theme comprised reasons that were specific to the personality or current situation of the recipient that affected helping decisions. The likelihood of helping increased when the recipient showed signs of wanting to change their circumstances, and play an active role in effecting that change. Attentive helpers were also aware of when recipients might be especially vulnerable. Knowledge of a person’s difficult circumstances motivated P10 and P12 to ask people close to them whether they were contemplating suicide, with P10 explaining, *“…a distressing event had occurred in the family, so this family member…was becoming distressed…And I was concerned for them, I knew that they were not taking it well…she was vulnerable and I was worried about her. And I cared about her, I didn’t want her to be distressed. And then I was worried about the threat to her life, when she did disclose that she was thinking about it, I wanted to make sure that she was safe…I wasn’t surprised that she started getting depressed, but I didn’t necessarily think that she was going to become suicidal.”*

Conversely, participants might avoid helping because they had previously tried to help the recipient with little or no success, or they believed that the recipient was not currently motivated to change their situation. Participants were reluctant to become involved when they believed that the recipient expected the helper to fix their problems for them, or that the recipient might become dependent on the helper to the detriment of the helper’s wellbeing. P9 and P16 noted that no matter how much help they provided, they could not effect change for the recipient solely through their own efforts. P9 said, *“It’s like, going back to the heroin addict example, I couldn’t make him quit. He knew that he wanted to get clean, I knew that I wanted him to get clean, everyone wanted him to get clean, and he didn’t do it. And I can’t make him do it….it might be a compromise responsibility at different points, but it’s still ultimately their responsibility.”*

Particular characteristics of the recipient or their mental health problem could deter helping, including being introverted and withdrawn from social activities (P12); refusing to discuss (P11) or obtain professional help for their problems (P9); not believing they had any mental health problems (P6); being rude, uncooperative, condescending or abusive (P6, P7, P12); and believing that the recipient would react negatively if the helper brought up their mental health problems (P10, P14, P16). Having the recipient refuse, not want or not appreciate help was another deterrent.

#### When should I approach?

Concepts in this theme included the presence and severity of the recipient’s symptoms and situation-specific factors.

#### Symptom presence and severity

Almost every participant said that simply knowing that the recipient was distressed or symptomatic influenced their helping decision. For some, this involved understanding that the person’s behaviour was out of the ordinary for them, that it was unacceptable or unusual behaviour in any context, or that particular symptoms had reached concerning levels. For others, being approached by the recipient or someone close to them encouraged action. P5 suggested that clear indicators or communication of a mental health problem or mental health crisis strengthened the idea that the situation required attention. She said that she had most often helped *“…when those friends can state that they’re having difficulty, and need to talk about it.”*

However, participants were less likely to help when they judged the recipient’s mental health problem to be of low severity or not urgent enough to warrant immediate attention. The consensus was that this would lead them to monitor the recipient for adverse changes or signs of crisis, rather than basing their decision solely on their initial observation. In this sense, the decision to help was contingent on when it would be appropriate and necessary to offer help. P11 offered a typical example of this strategy: *“And there was one kid who I started noticing because of his interactions with some of the other students…it would have been a few weeks of observation…I wouldn’t ever go off half*-*cocked. It would have been me gradually developing, um, some understanding that* [the student’s] *mental status wasn’t quite fitting in with normal parameters…*[until] *I thought I’d developed enough of a case to be able to not look ridiculous if they then called him in and found that he was playing games.”*

#### Situation-specific factors

This sub-theme related to aspects of a situation that affected helpers’ ability to offer or provide effective help. Physical proximity was occasionally seen as important in deciding whether or not to help, how much help to offer and how effective the help might be, with increasing distance generally believed to deter or reduce the efficacy of helping. Perceiving a potential helping situation to be ambiguous or uncertain also affected helping decisions. P5 said, *“Maybe in the instances where I do do something, it’s because there’s something recognisable…whereas…someone on the street…there’s no clue as to what the problem might be or what the scope of it might be.”* This type of uncertainty seemed to be an upfront deterrent, inhibiting any approach.

Participants suggested that some helping instances were simply a combination of availability and opportunity, or being in the right place at the right time. P13 and P14 also gave examples where their help was incidental to relationships that had been formed for other reasons; their assistance was just another, small aspect of the overall situation. In contrast, most participants said that they would not help if other people (friends, family, carers, police or mental health professionals) were already assisting a recipient, believing there was little else they could contribute. Sometimes the helper was not the recipient’s preferred source of help, although they could support the recipient, or the primary helper, in other ways (e.g., by contacting the preferred helper, or checking in regularly with other helpers).

Some participants said that indirect help (e.g., asking someone else who knew the recipient better to make the approach, or calling professional or emergency help) might be more effective for, or appreciated by, a recipient. P10 said that in the workplace there were fewer chances to appropriately approach. P14 explained that he may be unlikely to hear about a crisis affecting someone he was not close to until after its resolution, saying *“I don’t think it would be appropriate given the closeness of our relationship and the fact that he’s got a wife and his brother lives here now as well and he’s got, kind of, immediate family. Not to kind of handball responsibility to them, but I just feel like I probably wouldn’t be the first person he would call in that situation.”*

#### When considering reasons for involvement is skipped

Five narratives indicated that considering reasons for and against helping is skipped or only briefly acknowledged in emergency or crisis situations (represented by the dotted arrow on the right hand side of Fig. 1). Participants primarily focused on the urgency and seriousness of the situation, and they almost immediately engaged in helping actions like cleaning wounds or approaching an aggressor, without considering any other potentially relevant factors. Often the helper and the recipient shared a close relationship and the wellbeing of the recipient was foremost in their minds. P4 commented on the transcript that her decision to help her partner the first time she noticed cuts on her wrists was almost entirely emotionally driven, however, she also wrote, *“I don’t think I could have NOT helped in this situation. If this was a stranger I may not have gone to such intimate levels of help…But because I felt so intimately close to this person, I felt a real protective intuition…”* In contrast, P14 wrote on the transcript that his crisis assessment was more methodical: *“…At the time I was making those early decisions we didn’t know whether she’d attempted suicide or had just gone for a walk without telling anyone, so we didn’t want to escalate the whole thing…After we realised that she wasn’t in the house and her walking shoes were by the front door, the more rational help*-*provision thing kicks in (calling emergency services* etc*.)…”*

### Action taken by the helper

Participants discussed an extensive array of actions they undertook to address different situations, encompassing direct, indirect and no assistance. Participants perceived or defined helping in various ways and provided information that suggested different levels of direct help. For example, they spoke about providing routine assistance as part of an existing relationship (e.g., “catching up,” P14), actively engaging with the recipient in ways that utilised components of the MHFA action plan (P4), providing intensive levels of help and support during a crisis (P10), or becoming so involved with recipients that they either became overwhelmed or adopted caring roles for long periods of time (P5).

There were far fewer, and much less involved, discussions of not helping in the dataset. There was also diversity in the way “not helping” was interpreted. Some participants described situations where they had offered help to someone in the past, but later withdrawn that help when it was poorly received (P11) or the recipient became dependent on the helper to the detriment of the helper’s wellbeing (P4, P5). Another common interpretation involved providing indirect help by, for example, calling the police (P16) or asking others to assist instead (P9). The remaining examples involved times when participants had actively chosen not to assist someone who they believed was distressed and needed help. Thus, there were also different levels of not helping, or non-engagement, including actively refusing to help, monitoring or observing changes in the recipient over time and providing indirect help.

**Fig. 2:**
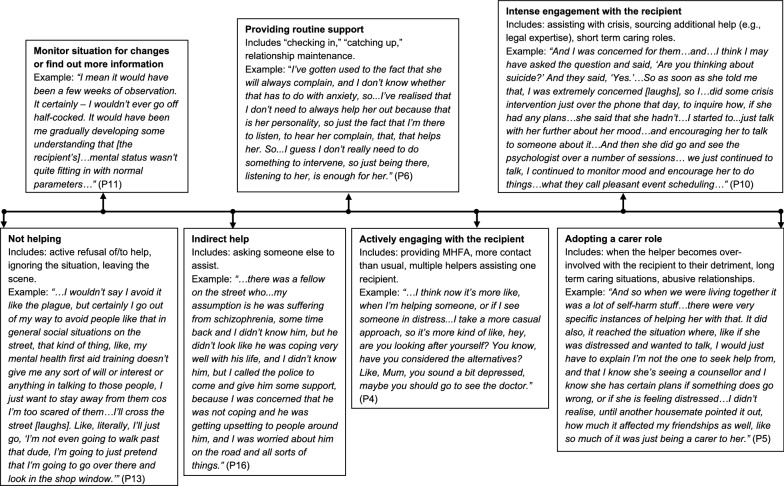
The helping spectrum: types of help and examples. The arrows indicate the locations of anchor descriptions and examples

The impression formed from participants’ helping experiences implied a sliding scale, or spectrum, of involvement, ranging from “refusing to help” to “providing too much help.” Participants’ narratives indicated that help could be adjusted according to the needs and circumstances of the helper and the recipient over time and across different helping situations with the same, or different, recipients. The spectrum also accounts for individual helper differences. For example, some helpers were often willing to assist strangers or acquaintances (e.g., P1); others could sustain long-term, intensive helping (e.g., P9). Figure 2 depicts the helping spectrum, with accompanying examples from participants to illustrate different helping and non-helping actions.

#### Multiple iterations of help

Participants involved in long-term helping situations often discussed specific helping incidents within the broader relationship context. Sometimes these were associated with different motivating factors, and often different actions were performed depending on the information available, the presenting symptoms or crisis, the needs of the recipient and the state of the helper at the time. This implies that if there is an ongoing relationship between the helper and the person, and the distress or problem is prolonged, helpers will cycle through recognising triggers to action, establishing whether they can and should help, and performing an action until the recipient improves or the helper decides to remove themselves from the situation. This is represented by the dotted arrow on the left hand side of Fig. 1.

### Outcomes of help

Participants frequently noted the impact that their help had on both the recipient and themselves, except when the help was ongoing and the situation had not yet resolved, or the helper was assisting strangers and never knew the outcome. The outcomes discussed include both the short- and long-term effects of help (e.g., the recipient feeling validated initially, and then recovering). Although details about the recipients’ outcomes were quite concise, they could be easily classified into positive, negative or mixed/unknown sub-themes. The outcomes of helping situations for participants fell into the same sub-themes, although an additional sub-theme was included, termed “a learning opportunity.” Participants were occasionally profoundly affected by their helping, perceiving it as an opportunity to learn more about themselves and/or others, and occasionally detailed how they had, or would, apply their newfound knowledge.

#### Outcomes for the recipient

Most helping situations were perceived to have beneficial outcomes for the recipient’s situation and wellbeing, for example, they received professional help and recovered, the helper’s intervention ceased the recipient’s unsafe or unhelpful behaviour, or the recipient’s condition was being managed. Occasionally the recipient would explicitly thank or express gratitude to the helper.

Help sometimes resulted in comparatively poor outcomes. For example, P11 explained that intervening indirectly to help a student who was developing schizophrenia resulted in his eventual hospitalisation and institutionalisation: *“He left school…at some stage in year 10, he was…put into care of some sort…I did bump into his mum at the supermarket every now and then and ask how he was. And so I’d get a, you know, ‘Not too good.’…he was at home, medicated, or he was somewhere else, medicated…It was a bad outcome, I felt sad for the bad outcome. Did I regret doing anything about it? No, not at all.”* P9 was pessimistic about her friend seeking help for or otherwise recovering from her increasingly severe depression. She stated that regardless of how much help she provided, her friend would not improve, because she was unlikely to accept the severity of her condition and change her behaviour now or in the future.

Sometimes the helper did not know what the recipient’s outcomes were or would be. As a telephone counsellor, P6 frequently had to accept that she would never know what happened to callers after the call ended. P15 described her brother’s disorder as requiring ongoing management and acknowledged that this situation would probably never have a clear outcome.

#### Outcomes for the helper

Participants discussed the positive effects of helping on their wellbeing or sense of self, explained how they felt about the resolution of the helping event, or described how they thought their actions were received by the person they helped. P1 said, *“I felt good, and it was a good outcome, he’d gotten off* [the tram]*, and we’d avoided a fight or anything bad…I felt like I’d restored the peace, so that was nice.”* P6 and P7, who both volunteer, perceived their efforts to be worthwhile and beneficial to them and their recipients. P2 said she had created a strong friendship with someone new. P10 said she primarily felt relief, and that she had done her best under the circumstances.

Negative outcomes associated with having provided help were also described, including feelings of uncertainty, regret, worry or frustration, or losing or scaling back a relationship with the recipient. P4 said that she was often unsure of whether she was really helping her recipients, and that sometimes she felt guilty about how she resolved helping situations. P6 talked about counselling calls that frustrated her, for example, when callers did not have a purpose or were disrespectful towards her or the service: *“…I’m just genuinely trying to help people who want help and then you’re wasting my time because you’re just* - *I don’t even know whether you want help…”*

Mixed feelings about helping were also discussed. P6 indicated that she was slowly becoming inured to people’s problems the longer she volunteered: *“So at first it was…really rewarding, but I find that I’m getting more desensitised…especially when you get regular callers, and they have the same problems, and you tell them the same thing each time and they don’t do anything about it. That’s when I feel desensitised and a bit helpless.”* P15 said she often felt like she had to suppress her frustration with her father, who has bipolar disorder, to maintain a relationship with him.

Several participants said they had learned valuable lessons from their helping experiences. These narratives generally involved a helping experience that profoundly affected the helper during or immediately after the event, which they processed over time and reinterpreted positively. P4 and P16 explained how ending very intense romantic relationships eventually resulted in redefining their personalities and philosophies. P16 said she had learned more about her capabilities and boundaries, and was now more selective about who she chose to assist. P4 said that she had learned the most about the importance of self-care and maintaining her independence within relationships.

### Member checking

The research findings were presented at an annual MHFA instructor conference in September 2015. This was followed by a discussion with instructors, focusing on whether the model was easily understood, applicable as a teaching tool, and whether it had implications for how they would teach MHFA. The model was well received by instructors, who commented that *“nobody is talking about how to help,”* (Instructor 9) and that the questions asked by the research were *“a bedrock of the program,”* (Instructor 12). Many instructors mentioned that while the findings were important, they would find it difficult to incorporate the model into the current structure of the course. Suggestions for incorporation included interactive scenario-based activities and creating a brief follow-up course to MHFA around *“how to help the helper help”* (Instructor 9) for interested participants. Instructors also discussed how to impart parts of these findings to their participants, for example, asking them to think about how they might react to a mental health crisis in a loved one before the situation occurs to avoid placing themselves in danger.

## Discussion

This study aimed to provide insight into the factors that motivate and deter adults from offering help to someone with a mental health problem and inform the development of a preliminary model of help giving which describes the process of providing help (Fig. 1). The model consists of five main steps: recognising a problem exists; considering reasons that might facilitate or deter helping; performing an action on the helping spectrum; and assessing the immediate and long-term effects of the helping action on the recipient and the helper. It also includes elements that address the cyclical nature of some helping interactions and how crises can cause the helper to jump from triggers to taking immediate action.

Although few studies have focused on the decision-making process surrounding helping someone with a mental health problem, previous research has identified some factors that facilitate and deter helping. A study of Australian university students found that most helped because they felt a duty as a friend, could relate to the peer’s concerns or feelings or were reciprocating previously provided help [26]. Participants in other research have cited reasons for not helping, including the recipient already receiving help or not wanting help [21, 24, 26, 27], believing it was not their place to help [26] and not being sure if help was required [17, 26]. These reasons closely resemble those mentioned by participants in this study, implying that they may be widely considered among the public. Similarly, studies that examined the outcomes of helping reported predominantly positive outcomes for helpers and recipients [21, 26]. While this study found many positive results of providing help, several narratives also noted poor or uncertain outcomes. The two studies mentioned above gathered their results from online surveys, so perhaps participants did not elaborate on their experiences to the extent that participants in these interviews did.

Owens et al. [17], in their study of people bereaved by suicide, explain that public health campaigns which encourage people to act when they see signs of a developing mental health problem in someone they know are premised on the idea of “If you see this, you should do that” [17; p.6]; however, in practice this is complicated by various respondent and recipient factors. The helping model presented here also illustrates this, and supports the suggestion that public education campaigns and training programs should address helper-centred and interpersonal barriers to intervening in mental health first aid situations wherever possible, for example, having open, sensitive discussions about mental health problems and emphasising the need to act on concerns around suicidality, even if this means contravening social norms such as privacy. Program developers might also consider incorporating elements of the helping model into scenario-based group discussions, or adapting the model into a decision aid, to encourage participants to consider what might be involved in assisting a person with a mental health problem prior to helping someone outside of the learning environment.

This research illustrates the insights that can be provided by using a qualitative approach to understanding the decisions and actions of individuals in mental health first aid situations. It offers a more detailed understanding of the dynamic, interactive nature of the helping process, the complex decisions involved, and how helpers and recipients influence each other’s perceptions of mental health, mental health problems and mental health first aid. The helping spectrum in particular is a new addition to the helping literature. It emphasises the fluidity and changeability of helping events and the importance of adjusting helping behaviours in response to the needs of the recipient, the situation and the helper. The model may be refined in future research and/or used as a basis for testing specific hypotheses. More broadly, this study’s findings concur with that of existing quantitative and qualitative literature, suggesting that the results may not be limited to this particular sample of respondents. The stages of the model also reflect elements of existing theories of helping behaviour, such as the theory of diffusion of responsibility in which individuals progress from noticing a problem to taking responsibility for helping and subsequently providing assistance [28].

Although many of the experiences mentioned by participants involved times they helped after doing the MHFA course, these examples do not comprise the majority of the data. Additionally, several participants discussed helping situations that they were involved in both before and after the course, or articulated how the course changed the way they helped a particular recipient over time. These results suggest that a reasonably diverse and comprehensive set of experiences were captured by choosing to use a sample of MHFA-trained community members. However, a small, purposive sample, who self-selected into this research, does reduce the transferability of the findings. Participants may have had particular characteristics, such as many prior helping experiences, which predisposed them to do the MHFA course or participate in this research. They therefore may have emphasised facilitators or deterrents to helping that differ from those of people who have less interest or training in mental health issues. Future research should interview participants with minimal or no experience or interest in helping people with mental health problems to explore how their reasons for helping differ from those of this sample. It is likely that there are motivators and deterrents that have been over- or under-represented in this research, or not mentioned at all (e.g., not recognising that a mental health problem is developing or present). Participants were recruited solely from one urban university and, despite the diversity in cultures and experiences, most were young, female and well educated. Future research could include older people, males, people living in rural areas and people from diverse educational backgrounds to broaden the applicability of the model and better understand the helping experiences of other groups. There were fewer non-helping situations discussed by participants, which limited the number and strength of the conclusions that can be drawn from this subset of the data. Furthermore, respondents may have experienced problems with recall, as many situations had begun or occurred years or months ago, and non-helping situations may have been forgotten.

As previously noted, the concepts of helping and not helping were quite subjective, and different people experiencing the same event may describe and interpret it very differently. Another limitation of this research could therefore be the absence of corroboration, or an alternative perspective, on the helping experiences recounted by helpers. For example, in the absence of data from the recipient, it remains unknown whether they perceived the help as beneficial or detrimental, and how the helper’s actions affected their subsequent outcomes. Research with people experiencing depression suggests that while their family and friends are often helpful, supportive, and caring, they can also lack compassion, knowledge and understanding [29]. It is therefore important for future research to connect with recipients of aid to more accurately and reliably assess the effects of the help provided by the first aider. Pairs of helpers and recipients could be interviewed about the same helping event to understand what each person gained or learnt from it. Program developers and evaluators may also find this information useful for understanding what recipients find helpful from their first aiders and how helpers can best approach and assist someone with mental health problems.

Two other limitations relate to the methodology of this study. Currently, it is not possible to articulate the relative importance or influence of the different considerations for becoming involved cited by participants. They remain as lists of factors, and only the frequency with which they were mentioned in the dataset indicates their importance more generally. A different investigation could focus on what people perceive to be the most compelling reasons for becoming involved using, for example, a card-sorting methodology as part of an interview [30] or a ranking task, if participants completed a survey. Establishing the predominant influences on helping behaviour would assist in refining the model and developing targeted mental health campaigns or education programs to directly address particular deterrents. Lastly, only one person coded the interview data, so interpretations were not checked or compared with those of other, independent raters, and this may affect the reliability of the findings. However, the coding process was discussed with the researchers supervising the project, guidance was sought from a qualitative researcher independent of the project prior to commencing coding, and member checking was performed with both participants and potential end users of the model.

## Conclusions

This study supports conclusions drawn from previous research into how members of the public assist a person experiencing mental health problems. It extends the existing literature on helping behaviours in several ways: by increasing the number of qualitative studies, and studies of helping behaviour, in this literature; by highlighting the complex interactions between helpers, recipients and the situations they encounter; and by providing a more comprehensive understanding of the process of helping, the reasons influencing offers of help, and the outcomes of helping. The preliminary model developed from this will hopefully prove useful for framing future research questions and studies.

## Abbreviation

MHFA: Mental Health First Aid
